# Microstructural Evolution and High-Performance Giant Dielectric Properties of Lu^3+^/Nb^5+^ Co-Doped TiO_2_ Ceramics

**DOI:** 10.3390/molecules26227041

**Published:** 2021-11-22

**Authors:** Noppakorn Thanamoon, Narong Chanlek, Pornjuk Srepusharawoot, Ekaphan Swatsitang, Prasit Thongbai

**Affiliations:** 1 Giant Dielectric and Computational Design Research Group (GD–CDR), Department of Physics, Faculty of Science, Khon Kaen University, Khon Kaen 40002, Thailand; kaopunkumpun@gmail.com (N.T.); spornj@kku.ac.th (P.S.); ekaphan@kku.ac.th (E.S.); 2 Synchrotron Light Research Institute (Public Organization), 111 University Avenue, Muang District, Nakhon Ratchasima 30000, Thailand; narong@slri.or.th

**Keywords:** giant/colossal permittivity, TiO_2_, impedance spectroscopy, temperature coefficient, IBLC

## Abstract

Giant dielectric (GD) oxides exhibiting extremely large dielectric permittivities (ε’ > 10^4^) have been extensively studied because of their potential for use in passive electronic devices. However, the unacceptable loss tangents (tanδ) and temperature instability with respect to ε’ continue to be a significant hindrance to their development. In this study, a novel GD oxide, exhibiting an extremely large ε’ value of approximately 7.55 × 10^4^ and an extremely low tanδ value of approximately 0.007 at 10^3^ Hz, has been reported. These remarkable properties were attributed to the synthesis of a Lu^3+^/Nb^5+^ co-doped TiO_2_ (LuNTO) ceramic containing an appropriate co-dopant concentration. Furthermore, the variation in the ε’ values between the temperatures of −60 °C and 210 °C did not exceed ±15% of the reference value obtained at 25 °C. The effects of the grains, grain boundaries, and second phase particles on the dielectric properties were evaluated to determine the dielectric properties exhibited by LuNTO ceramics. A highly dense microstructure was obtained in the as-sintered ceramics. The existence of a LuNbTiO_6_ microwave-dielectric phase was confirmed when the co-dopant concentration was increased to 1%, thereby affecting the dielectric behavior of the LuNTO ceramics. The excellent dielectric properties exhibited by the LuNTO ceramics were attributed to their inhomogeneous microstructure. The microstructure was composed of semiconducting grains, consisting of Ti^3+^ ions formed by Nb^5+^ dopant ions, alongside ultra-high-resistance grain boundaries. The effects of the semiconducting grains, insulating grain boundaries (GBs), and secondary microwave phase particles on the dielectric relaxations are explained based on their interfacial polarizations. The results suggest that a significant enhancement of the GB properties is the key toward improvement of the GD properties, while the presence of second phase particles may not always be effective.

## 1. Introduction

An effort to develop giant dielectric (GD) materials has been driven by an increased demand for high-energy-density storage devices in the electronic industry [1]. In the case of dielectric applications, such as ceramic capacitors, a high dielectric permittivity material exhibiting a dielectric permittivity (ε’) greater than 10^3^ and a low loss tangent (tanδ < 0.025) is required to reduce the component’s dimensions by increasing the ε’ value exhibited by the dielectric layer. Moreover, the GD materials should exhibit stable dielectric properties with respect to the temperature and frequency over a broad range of conditions.

Recently, a significant number of GD materials have been developed, including CaCu_3_Ti_4_O_12_ (CCTO) and related compounds [2,3,4,5], CuO [6], La_2−x_Sr_x_NiO_4_ [7], and NiO-based groups [8]. Owing to the significant research in this field, the dielectric mechanisms in these materials are clearly understood. However, because of the high tanδ values exhibited by these materials, they cannot be utilized for practical applications. Furthermore, the ε’ exhibited by these materials is strongly dependent on their temperature, a dependence which needs to be eliminated to ensure their widespread application in the future.

Recently, a novel elegant GD material, specifically In^3+^/Nb^5+^ co-doped TiO_2_, was reported to exhibit an ε’ > 10^4^ and a tanδ < 0.05 [9]. Furthermore, this material exhibited stable dielectric properties with respect to the frequency and temperature across a wide range of values. The large concentration of the induced defect-clusters ($In_{2}^{3+}V_{0}^{••}Ti^{3+}$ and$\;Nb_{2}^{5+}Ti^{3+}M_{Ti}$ ($M=Ti^{3+},Lu^{3+},Ti^{4+}$)), known as the electron-pinned defect-dipoles (EPDDs), has been suggested to be imparting the dominating mechanism within this material. Subsequently, the GD properties exhibited by the TiO_2_-based materials consisting of numerous co-doping systems had been extensively evaluated, including rutile-TiO_2_ co-doped with several +1/+5, +2/+5 components and other +3/+5 co-dopant systems [9,10,11,12,13]. Accordingly, several mechanisms, including electron hopping, the internal barrier layer capacitor (IBLC) model, the surface barrier layer capacitor model, and a compositional gradient resulting in the formation of a local structure, were proposed to explain the GD properties exhibited by these co-doped TiO_2_ systems [14,15,16,17,18]. Depending on the various co-doping elements, several mechanisms contribute to the dielectric phenomena exhibited by the co-doped TiO_2_ system.

One of the most interesting co-dopants is ***Ln***^3+^/Nb^5+^ (or Ta^5+^), where ***Ln*** = La, Dy, Pr, Nd, Eu, Er, Gd, and Sm [19,20,21,22,23,24,25,26,27,28,29,30]. Owing to the large ionic radii (*r_6_*) exhibited by the ***Ln***^3+^ ions, when they are substituted into the rutile-TiO_2_ structure, EPDDs may subsequently be induced [26,31]. In addition, most of these defects exhibit very large ε’ values of approximately 10^4^–10^5^, and relatively small tanδ values. Moreover, owing to the fact that **Ln** elements exhibit significantly larger ionic radii than their surrounding elements, a number of studies have reported the formation of second-phase particles through ***Ln***^3+^/Nb^5+^ (or Ta^5+^) co-doping in TiO_2_. This process was suggested to impart the reduced tanδ values [21]. Considering this, the ***Ln*** elements form some of the most interesting acceptor ions for utilization as dopants in co-doped TiO_2_ systems. Hu et al. [32] reported a high ε’ and low tanδ in the (Nb^5+^, Lu^3+^) co-doped TiO_2_. However, the effects of the microstructure evolution and second phase particles on the GD response and electrical properties of the grain and grain boundary for this co-doped TiO_2_ system have never been reported. To clearly describe the origin of the GD properties, impedance spectroscopy must be performed.

In this study, we firstly report the influences of microstructure and second phase particles on the GD properties of a co-doped TiO_2_ system of Lu^3+^/Nb^5+^ (LuNTO) ceramics. Impedance spectroscopy was used to separate the electrical responses of the semiconducting and insulating parts. Owing to the larger ionic radii exhibited by both Lu^3+^ (86.1 pm) and Nb^5+^ (64.0 pm) in comparison to that of Ti^4+^ (60.5 pm), the GD properties exhibited by these materials may be attributed to several factors including EPDD, IBLC, and secondary-phase particles. The LuNTO ceramics were prepared via a solid-state reaction (SSR) process. The highest dielectric performance exhibited by a LuNTO ceramic recorded a very high ε’ value of approximately 7.5 × 10^4^, while also exhibiting excellent temperature stability between 60 °C and 210 °C and a very low tanδ value of approximately 0.007. The tanδ value exhibited at 200 °C (approximately 0.05) was also acceptable.

## 2. Results and Discussion

### 2.1. Crystal Structure and Phase Compositions

**Figure 1:**
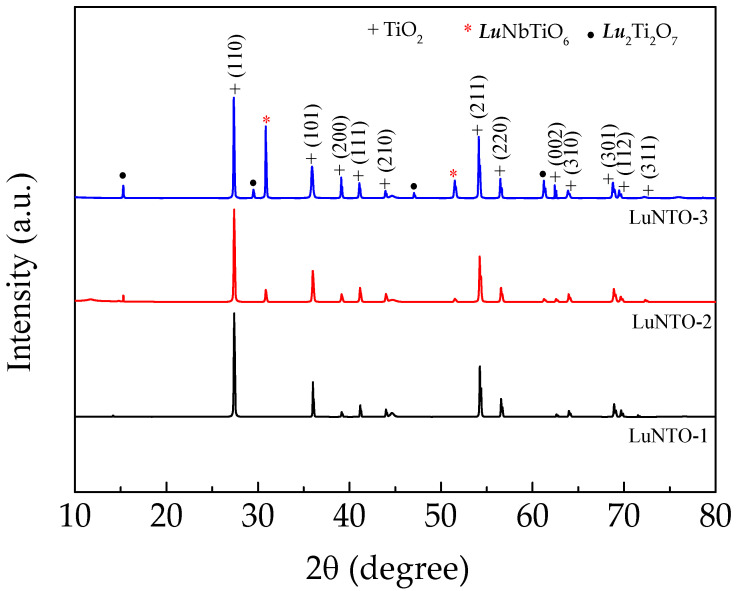
XRD patterns of the LuNTO ceramics.

Figure 1 shows the XRD patterns obtained from the LuNTO ceramics containing different co-dopant concentrations, ranging from 0.5–2.5%. The XRD spectra obtained from each of the LuNTO ceramics were consistent with those obtained from the main phase of rutile TiO_2,_ adopting a *P*4_2_/*mnm* space group, i.e., a tetragonal structure (JCPDS 21-1276) [33]. The lattice parameters (*a* and *c* values) are listed in Table 1. Owing to the larger ionic radii (*r*_6_) exhibited by the dopants (86.1 pm and 64.0 pm for Lu^3+^ and Nb^5+^, respectively), the lattice parameters of the LuNTO ceramic were larger than those of TiO_2_ (*r*_6_ (Ti^4+^) = 60.5 pm). The *a* and *c* values of the LuNTO ceramics tended to increase with increasing co-dopant concentration. The Lu^3+^ and Nb^5+^ dopant ions could either be partially or entirely substituted into the TiO_2_ structure. The impurity microwave-dielectric phase, ***RE***NbTiO_6_ (***RE*** = Lu), was observed in the XRD spectra obtained from the LuNTO-2 and LuNTO-3 ceramics [34,35]. A small quantity of an additional Lu_2_Ti_2_O_7_ impurity phase was also observed in the LuNTO-3 ceramic [36]. These microwave-dielectric phases typically exhibit a very low tanδ value and low conductivity [34,35,37,38]. The Lu^3+^ dopant ions could partially replace the host Ti^4+^ sites in the structure, while excessive Lu^3+^ ions are able to react with Ti^4+^ to form the microwave-dielectric phases.

### 2.2. Microstructure Analysis

**Figure 2:**
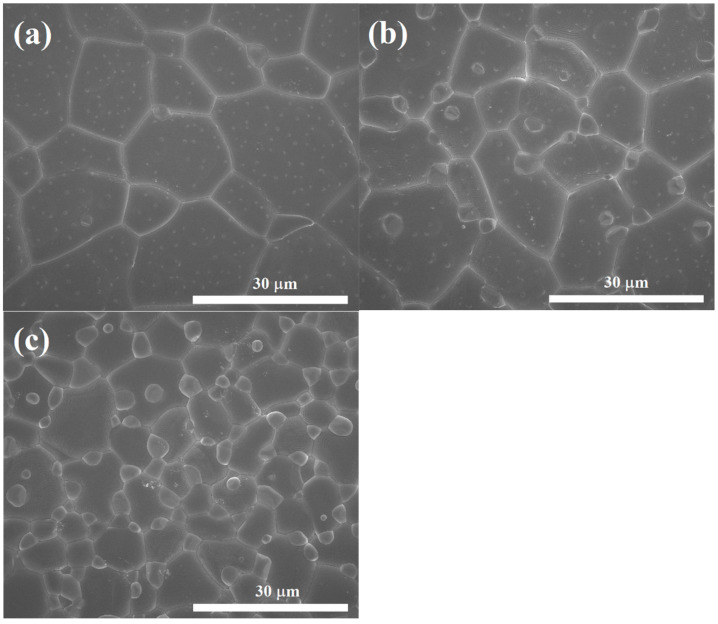
Surface morphologies of (**a**) LuNTO-1, (**b**) LuNTO-2, and (**c**) LuNTO-3 ceramics.

Generally, the dispersion of the second-phase particles throughout the ceramic matrix can influence the electrical and dielectric properties exhibited by the composites. As shown in Figure 2, all the LuNTO ceramics exhibited a dense microstructure without any porosity. The mean grain sizes for all the ceramics were summarized in Table 1. The mean grain size decreased with an increase in the co-dopant concentration, corresponding to the increased volume of these second phase particles. When the co-dopant concentration increased to 1.0% (*x* = 0.01), the small particles that are expected to be a second phase started to precipitate.

**Figure 3:**
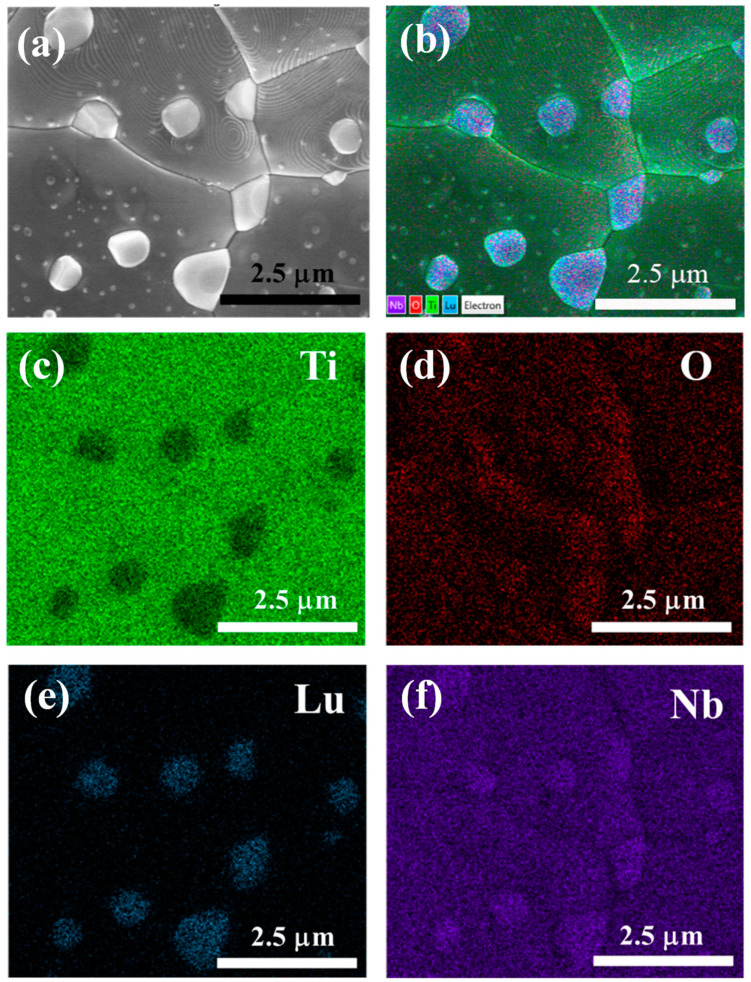
SEM-EDS mapping images of LuNTO-2 ceramic; (**a**) SEM image for mapping area, (**b**) all elements, (**c**) Ti, (**d**) O, (**e**) Lu, and (**f**) Nb.

**Figure 4:**
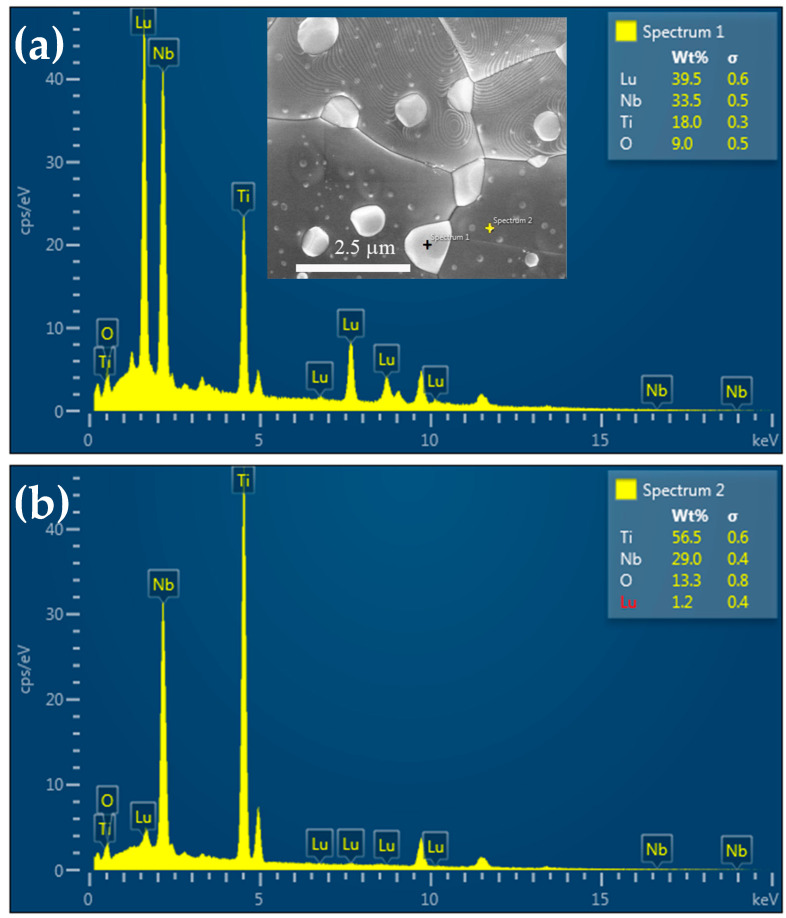
EDS spectra of LuNTO-2 ceramic detected at (**a**) the secondary phase particle and (**b**) regular grain; insets show the SEM images of the EDS testing points.

Figure 3 shows the elemental distribution of each element within the LuNTO-2 ceramic obtained via SEM-EDS mapping. Both Nb and O were homogeneously dispersed. However, both Lu and Ti were found to be non-homogeneously dispersed as small grain particles throughout the microstructure. The small grain particles are likely to form the second phase. According to the XRD analysis, only the LuNbTiO_6_ phase was detected in the LuNTO-2 ceramic, confirming that the second phase particles detected in the SEM mapping image correspond to the LuNbTiO_6_ phase. Figure 4a,b show the EDS spectra obtained from the grains and the secondary phase particles (as shown in the inset of Figure 4a, respectively. Lu, Nb, and Ti were all detected in the secondary phase particles, confirming the formation of the LuNbTiO_6_ phase. Generally, the LuNbTiO_6_ ceramic is classified as a microwave ceramic material within the ***RE***NbTiO_6_ classification, which typically exhibits an ultra-low loss tangent and high resistivity [34,35]. The LuNbTiO_6_ ceramic particles may exert a significant influence on the electrical and dielectric properties exhibited by the LuNTO ceramics. The presence of the secondary ***RE***NbTiO_6_ and ***RE***_2_Ti_2_O_7_ phases has previously been reported in other co-doped TiO_2_ systems, such as Dy^3+^/Nb^5+^ [21], (Y^3+^/Yb^3+^/Sm^3+^/Gd^3+^)/Nb^5+^ [20,39], Pr^3+^/Nb^5+^ [40], La^3+^/Nb^5+^ [26], Sm^3+^/Ta^5+^ [30], and Yb^3+^/Ta^5+^ [29].

The decrease in the mean grain size could be attributed to the pinning effect imparted by the second phase LuNbTiO_6_ particles [41]. During the sintering process, the mobility of the grain boundary (GB), which is a result of the diffusion of charge species from one grain to another, was inhibited by the LuNbTiO_6_ secondary-phase particles, resulting in a decreased grain growth rate for the LuNTO ceramics. According to the Zener model [41], the limiting grain size (*G*_*L*_) exhibited by the polycrystalline ceramics is directly dependent on the particle size of the second-phase particles (*r*) and inversely proportional to the volume fraction of the filler particles, following the relationship $G_{L}\propto \frac{r}{f}$. Thus, a continuous decrease in the mean grain size of the LuNTO ceramics of different co-dopant concentrations results in an increase in the volume fraction of the LuNbTiO_6_ particles.

### 2.3. Raman and XPS Spectroscopies

**Figure 5:**
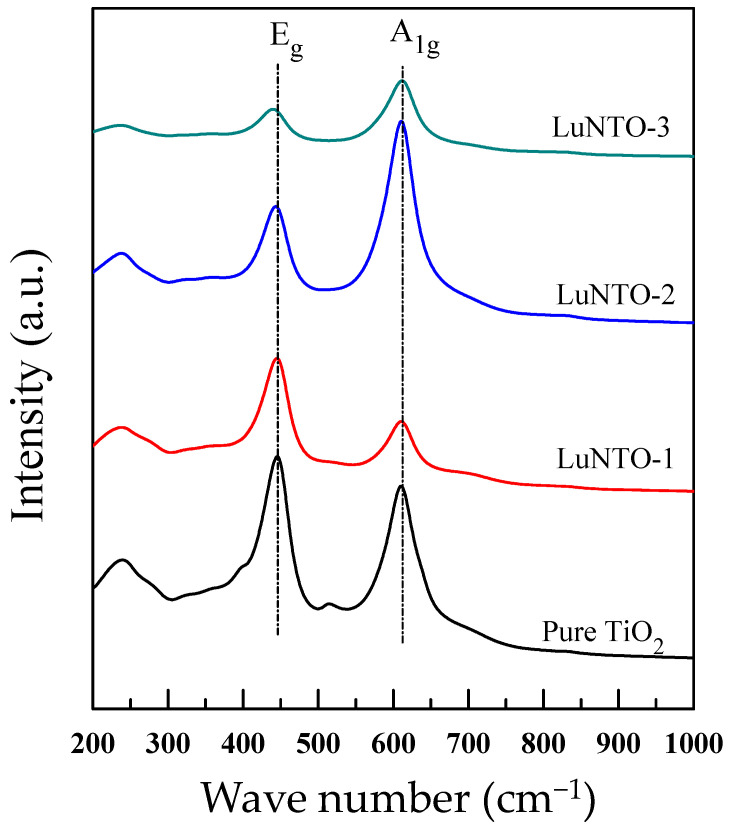
Raman spectra of TiO_2_ and LuNTO ceramics with various co-doping concentrations.

Figure 5 shows the Raman spectra obtained from the TiO_2_ powder and each of the LuNTO ceramics. Two primary modes were observed, i.e., *E*_g_ and *A*_1g_, in the range of 200–1000 cm^−1^. Through a comparison with the Raman spectrum obtained for the TiO_2_ powder (*E*_g_ ≈ 345.5 cm^−1^ and *A*_1g_ ≈ 610.5 cm^−1^), the *E*_g_ peaks exhibited by the LuNTO ceramics experienced a significant shift to a lower wavenumber as the co-dopant concentration increased, particularly in the case of the LuNTO-3 ceramic (*E*_g_ ≈ 339.0 cm^−1^). This result is indicative of the oxygen ion-induced lattice distortion along the *c*-axis [17,26,42]. Typically, an O vacancy is generated in the rutile-TiO_2_ structure by replacing a Ti^4+^ ion with an acceptor dopant ion such as Lu^3+^ for charge compensation and adheres to the following equation:

(1) $${\mathrm{Lu}}_{2}O_{3}+2{\mathrm{TiO}}_{2}\to 2Lu{’}_{\mathrm{Ti}}+2V_{O}^{••}+3O_{2}$$

Although the second phase adopted by the Lu-related phase particles was detected in both the XRD spectra and SEM images, the Raman spectroscopy results indicated that some portion of Lu^3+^ was substituted into the TiO_2_ structure. In contrast, the *A*_1g_ peaks corresponding to the O–Ti–O bond bending showed a slight fluctuation, for instance, *A*_1g_ ≈ 611.5 cm^−1^ in the LuNTO-3 ceramic.

**Figure 6:**
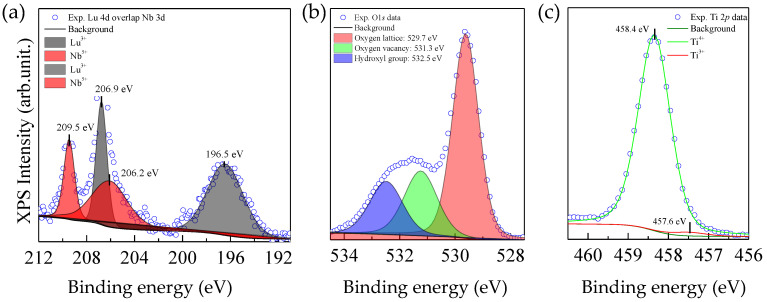
XPS spectra of LuNTO-3 ceramic; (**a**) Lu *4d* and Nb 3*d*, (**b**) O 1*s*, and (**c**) Ti 2*p*.

The XPS spectra obtained from Nb 3*d* and Lu 4*d* are shown in Figure 6a. Nb 3*d*_5/2_ overlapped Lu 4*d*_3/2_. The presence of Nb^5+^ was confirmed by the appearance of two peaks exhibiting binding energies of 209.5 eV and 206.9 eV, which corresponded to Nb 3*d*_3/2_ and Nb 3*d*_5/2_, respectively. The spin-orbit splitting exhibited by these two peaks was 2.6 eV, which is typically detected in Nb^5+^-doped TiO_2_ [9]. The XPS peaks detected at binding energies of 206.2 eV and 196.5 eV, corresponded to Lu 3*d*_3/2_ and Lu 3*d*_5/2_, respectively, confirming the presence of Lu^3+^ in the LuNTO ceramic [43]. As shown in Figure 6b, the O 1*s* profile exhibited three energy peaks at 532.5 eV, 531.3 eV, and 529.7 eV, which correspond to the O lattice (Ti–O bond), O vacancy, and hydroxyl group, respectively. According to the Raman spectroscopy, the O vacancy concentration in the LuNTO ceramics increased in comparison to the undoped TiO_2_ ceramic. The O vacancies were likely imparted by the Lu^3+^ dopant ions in accordance with Equation (1) and additionally by the O loss during high temperature sintering. In Figure 6c, the XPS spectrum obtained from Ti 2*p* was separated into two distinct peaks with corresponding binding energies of 458.4 eV (assigned as Ti^4+^) and 457.6 eV (assigned as Ti^3+^), respectively. The presence of a small quantity of Ti^3+^ was attributed to the substitution of Ti^4+^ with donor Nb^5+^ ions according to the following equations [9,15,44]:

(2) $$2TiO_{2}+Nb_{2}O_{5}\overset{4TiO_{2}}{\to }2Ti{’}_{Ti}+2Nb_{Ti}^{•}+8O_{o}+1/2O_{2},$$

(3) $$Ti^{4+}+e^{-}\to Ti^{3+}$$

According to the above results, the complex defect structures of $Lu_{2}^{3+}V_{0}^{••}Ti^{3+}$ and $Nb_{2}^{5+}Ti^{3+}M_{Ti}$ ($M=Ti^{3+},Lu^{3+},Ti^{4+}$) may be induced in the LuNTO ceramic. In contrast, the presence of Ti^3+^ may impart semiconducting grains, which can induce interfacial polarization at the internal insulating interfaces, such as GBs and secondary-phase particles.

### 2.4. Giant Dielectric Properties

**Figure 7:**
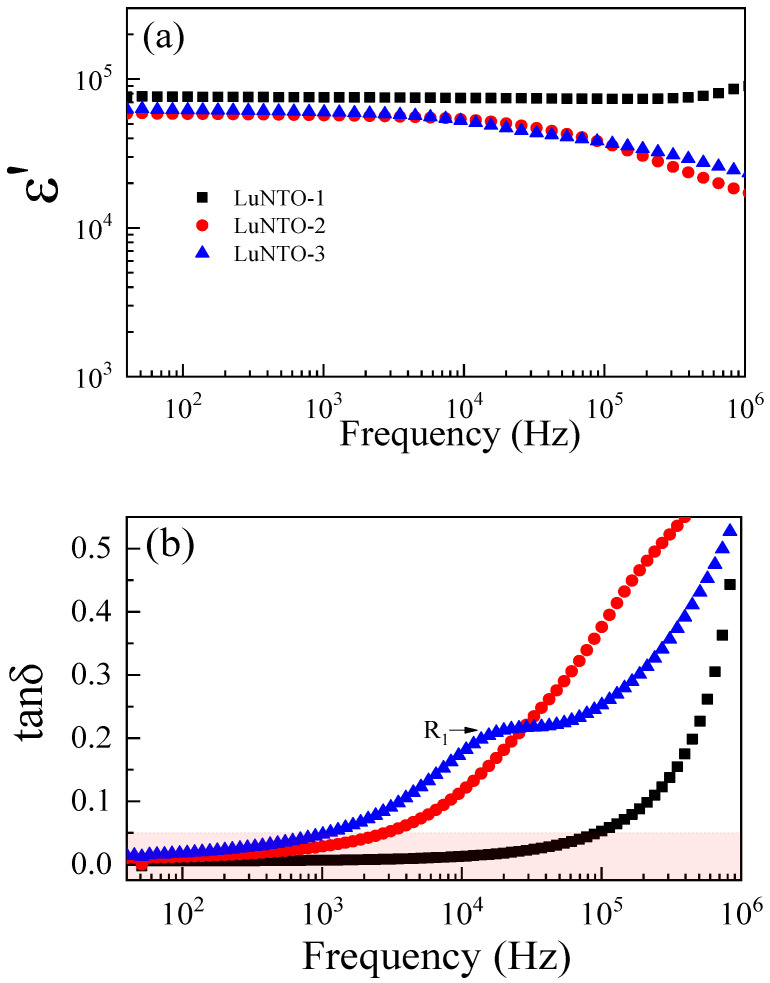
(**a**) Dielectric permittivity (ε’) and (**b**) loss tangent (tanδ) at 30 °C for LuNTO ceramics in the frequency range of 40–10^6^ Hz.

The LuNTO-1 ceramic exhibits the largest ε’ value (approximately 7.55 × 10^4^ at 1 kHz) over the measured frequency range, as shown in Figure 7a. In addition, between 40 Hz and 10^6^ Hz, ε’ is essentially independent of the frequency. As shown in Figure 7b, the tanδ value exhibited by the LuNTO ceramic does not exceed 0.05 in the frequency range from 40 Hz to 10^5^ Hz. Notably, the tanδ value exhibited by the LuNTO-1 ceramic at 1 kHz was as low as 0.007. These results are comparable to those previously reported in the literature with respect to In^3+^/Nb^5+^ and In^3+^/Ta^5+^ co-doped TiO_2_ [45,46]. Co-doping TiO_2_ with a small volume (0.5 at.%) of Lu^3+^ + Nb^5+^ imparts a significant improvement in the GD properties.

The LuNTO-2 and LuNTO-3 ceramics exhibit lower ε’ values in comparison to the LuNTO-1 ceramic across the measured frequency range. Furthermore, the frequency dependence with respect to ε’ exhibited by these two ceramics is more pronounced when the frequency exceeds 10^4^ Hz. The ε’ and tanδ values at 1 kHz at approximately 25 °C are listed in Table 1. The increase in tanδ at high frequencies is related to the concurrent decrease in the ε’. The primary tanδ peaks obtained from each of the ceramics, which corresponded to the primary polarization that imparted the high ε’, occurred at a frequency greater than 10^6^ Hz, despite the fact that a secondary tanδ peak (R1) was observed within the LuNTO-3 ceramic at ~10^4^ Hz. As shown in Figure 2, Figure 3 and Figure 4, a significant number of LuNbTiO_6_ particles were observed in the LuNTO-3 ceramic. Thus, the relaxation of the tanδ peak may be attributed to these particles, giving rise to the highest tanδ value. The tanδ values exhibited by the LuNTO-2 and LuNTO-3 ceramics at 1 kHz are higher than that by LuNTO-1 but still quite low in general. However, the values obtained can still be considered suitable for practical applications. The LuNTO ceramics containing a co-dopant concentration ≤ 2.5% exhibit GD properties, which indicates their potential in capacitor applications. The ε’ values were found to reduce as the concentration of the secondary particles increased, owing to the low ε’ of the LuNbTiO_6_ phase [35]. This result was in accordance with the mixed rule of two-dielectric phase composites [47]. The material can be described as a ceramic composite, composed of the high-permittivity LuNTO phase alongside the low-permittivity LuNbTiO_6_ phase.

**Figure 8:**
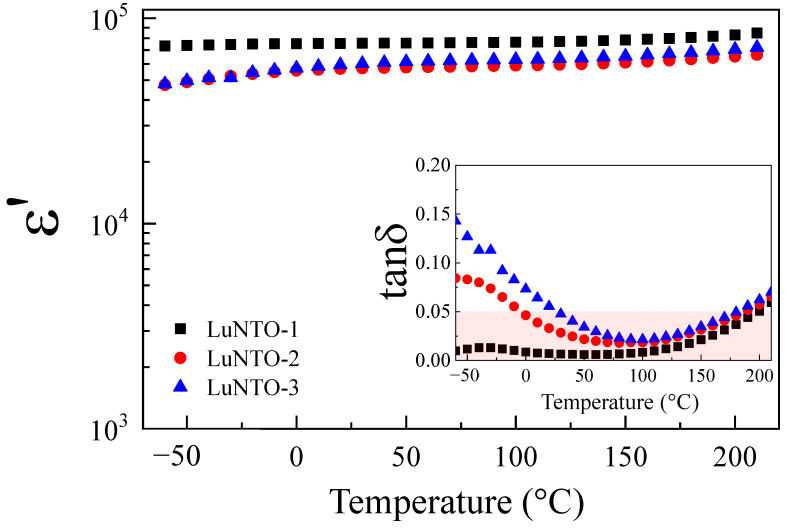
Dielectric permittivity (ε’) at 1 kHz as a function of temperature; inset shows the temperature dependence of the loss tangent (tanδ) at 1 kHz.

**Figure 9:**
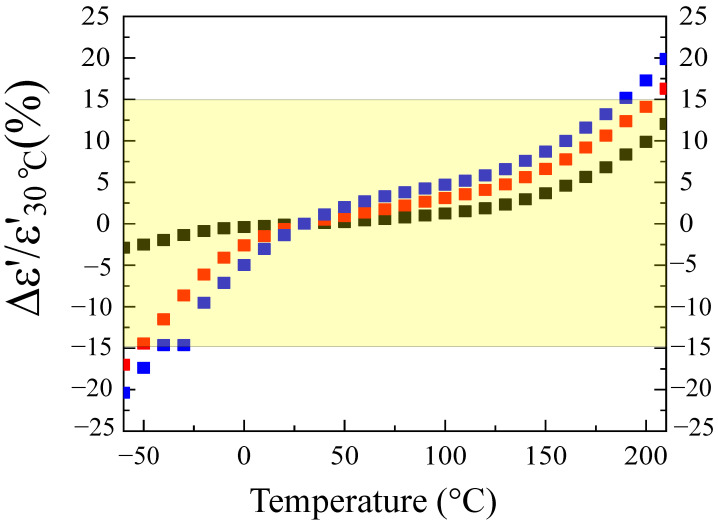
Temperature coefficient of ε’ at 1 kHz for all the ceramics.

Figure 8 illustrates the temperature dependence with respect to the dielectric properties exhibited by the LuNTO ceramics. Interestingly, each of the ceramics exhibited high temperature stability with respect to ε’, particularly in the case of the LuNTO-1 ceramic. Surprisingly, as shown in the inset in Figure 8, the LuNTO ceramics retained a low tanδ at 200 °C (approximately 0.050, 0.057, and 0.062 for LuNTO-1, LuNTO-2, and LuNTO-3, respectively). The tanδ value exhibited by the LuNTO-1 ceramic did not exceed 0.05 in the temperature range of −60 °C to 210 °C. The temperature variation of ε’ (1 kHz), also known as the temperature coefficient of ε’, in comparison to the values obtained at 30 °C (${\varepsilon^{\prime }}_{30{}^{\circ}C}$) was calculated for each ceramic according to the following equation: $\Delta \varepsilon^{\prime }\left(\%\right)=[{\varepsilon^{\prime }}_{T}-{\varepsilon^{\prime }}_{30{}^{\circ}C}/{\varepsilon^{\prime }}_{30{}^{\circ}C}]\times 100$, where ${\varepsilon^{\prime }}_{T}$ corresponds to the ε’ value at a given temperature, *T*. As shown in Figure 9, the temperature coefficient of the LuNTO-1 ceramic in the temperature range of −60 °C to 210 °C did not exceed ±15%. Notably, the LuNTO-1 ceramic exhibited a low tanδ value of approximately 0.007 at 1 kHz alongside a high ε’ of approximately 7.55 × 10^4^, where $\Delta \varepsilon^{\prime }{\varepsilon^{\prime }}_{30{}^{\circ}C}/\left(\%\right)\;\leq \pm 15\%$ in the temperatures between −55 °C and 200 °C, thereby meeting the basic requirement for its application in an X9R-type ceramic capacitor. These results are extremely hard to replicate in other varieties of GD oxides, including CCTO and other related compounds [2,48,49], CuO [6], co-doped NiO [50], and La_2−x_Sr_x_NiO_4_ ceramics [7]. Furthermore, the excellent dielectric parameters exhibited by the LuNTO-1 ceramics form one of the most interesting ***Ln***^3+^/Nb^5+^ (or Ta^5+^) co-doped TiO_2_ systems [22,23,26,28,29,30]. These values are comparable to the ones reported in other ***Ln***^3+^/Nb^5+^ (Ta^5+^) co-doped TiO_2_ systems, such as Gd^3+^/Nb^5+^ (tanδ ≈ 0.027 and ε’ ≈ 5.6 × 10^4^) [39], La^3+^/Nb^5+^ (tanδ ≈ 0.019 and ε’ ≈ 2 × 10^4^) [22], Eu^3+^/Nb^5+^ modified with B_2_O_3_ (tanδ ≈ 0.012 and ε’ ≈ 4.1 × 10^4^) [23], Nd^3+^/Ta^5+^ (tanδ ≈ 0.008 and ε’ ≈ 8.2 × 10^4^) [25], Dy^3+^/Nb^5+^ (tanδ ≈ 0.078 and ε’ ≈ 6.4 × 10^4^) [21], and Pr^3+^/Nb^5+^ (tanδ ≈ 0.037–0.075 and ε’ ≈ 6–8 × 10^4^) [40]. Among these co-doped TiO_2_ systems, only the LuNTO-1 (Lu^3+^/Nb^5+^) and Eu^3+^/Nb^5+^ systems modified with B_2_O_3_ exhibit a suitably low temperature coefficient of Δε’ < ±15% up to 200 °C.

### 2.5. Origin of High-Performance GD Properties

To evaluate the origin of the GD properties exhibited by the LuNTO ceramics, impedance spectroscopy was used to probe the electrical heterogeneity exhibited by the LuNTO ceramics. Generally, a large semicircular arc of the complex impedance plane (Z*) plot of most GD oxides (e.g., CCTO) can be observed at 25 °C in the frequency range of 10^2^–10^6^ Hz. This arc corresponds to the electrical response of the insulating regions, such as the GBs and/or the insulating outer layer [14,15,16]. Simultaneously, a nonzero intercept can also be observed, which corresponds to the electrical response exhibited by the semiconducting grains [11,17,18]. The resistance exhibited by the grains (R_g_) can be calculated from the nonzero intercept. In a number of cases, the large semicircular arc corresponding to the GB response is not observed at 25 °C owing to the significant total resistance imparted by the insulating regions within the material [10,17,18,51,52]. This issue can be resolved by increasing the temperature of the system to decrease the resistance of the GBs.

**Figure 10:**
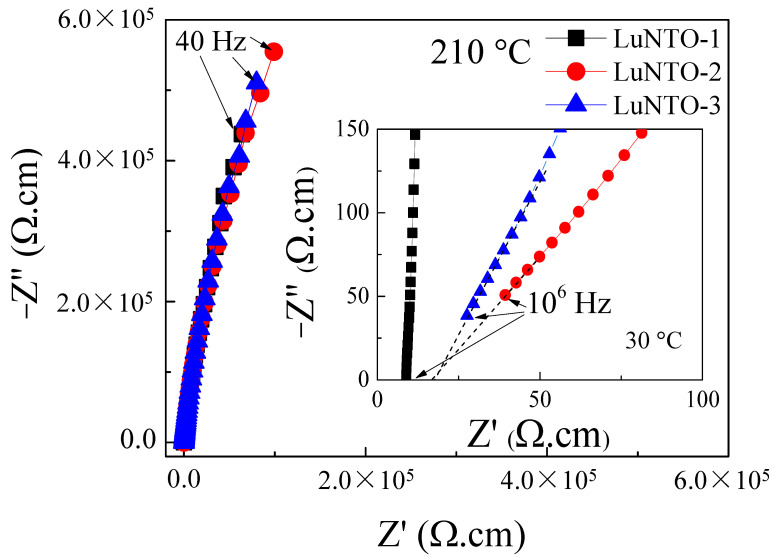
Impedance complex plane (Z*) plots at 200 °C for LuNTO-1, LuNTO-2, and LuNTO-3 ceramics; inset shows the nonzero intercept at high frequencies at 30 °C.

In this study, the upper temperature limit for the instrument was 210 °C. As shown in Figure 10, the large semicircular arc was not observed in the frequency range of 40–10^6^ Hz; even at 210 °C, only segments of the characteristic semicircular arc were observed. The total resistance exhibited by the insulating regions within each of the LuNTO ceramics is substantial across the entire measured temperature range. At 210 °C, the total resistance exhibited by the LuNTO ceramics is estimated to be greater than 10 MΩ·cm, which is much larger than the ones exhibited by GD oxides, such as CCTO (<5 × 10^4^ Ω·cm at 200 °C) [3,4], V^3+^/Ta^5+^ co-doped TiO_2_ (approximately 1.5 MΩ·cm at 150 °C) [10], Al^3+^/Ta^5+^ co-doped TiO_2_ (approximately 0.3 MΩ·cm at 200 °C) [52], and Gd^3+^/Nb^5+^ co-doped TiO_2_ (approximately 5 × 10^4^ Ω·cm at 150 °C) [39]. Inset of Figure 10, the nonzero intercept of the Z* plots for each of the LuNTO ceramics can be determined, indicating the presence of the semiconducting grains. Thus, the microstructure of the LuNTO ceramics consists of insulating regions exhibiting ultra-high resistivity, alongside semiconducting grains. The origin of the GD properties is primarily attributed to the IBLC structure. Nevertheless, it has been suggested, but not yet proven, that the EPDD effect may exert an influence on the GD properties exhibited by the LuNTO ceramics, since the ionic radius of the Lu^3+^ ions is sufficient (in comparison to In^3+^) to theoretically induce the formation of EPDDs.

The extremely low tanδ value of approximately 0.007 exhibited by the LuNTO-1 ceramic at 1 kHz and 30 °C is attributed to the ultra-high resistivity exhibited by the internal insulating regions, i.e., the GBs and secondary-phase particles corresponding to the LuNbTiO_6_ microwave-dielectric phase. The origin of the semiconducting grains is attributed to the Nb^5+^ doping ions, in accordance with Equations (2) and (3). Furthermore, the introduction of O vacancies during the high-temperature sintering process can also be attributed to the presence of the semiconducting grains. The tanδ values obtained at 1 kHz and 30 °C in the LuNTO-2 and LuNTO-3 ceramics were larger than the corresponding values obtained for the LuNTO-1 ceramic. Despite this, their resistivity values remained extremely large, as was the case in the LuNTO-1 ceramics. This result may be attributed to the existence of tanδ dielectric relaxation peaks at 10^6^ and 10^4^ Hz in the LuNTO-2 and LuNTO-3 ceramics, respectively (Figure 7b). The dispersion of the LuNbTiO_6_ microwave-dielectric phase particles was observed throughout the microstructure. Therefore, the interfacial polarization relaxation that occurs at the interface between the semiconducting LuNTO grain and the adjacent insulating LuNbTiO_6_ particles is induced. Generally, the significant increase in the tanδ value at high temperature is attributed to the long-range motion of free charge carriers or DC conduction [8], which can be effectively inhibited through an increase in the total resistance exhibited by the internal insulating layer. Consequently, the extremely large resistivity values exhibited by each of the LuNTO ceramics are the primary cause of the suppression of their tanδ values at high temperatures. This explanation is justified, as in the temperature range of 100–210 °C, the tanδ values exhibited by each ceramic underwent only a slight variation; in contrast, the tanδ values obtained at low temperatures exhibited a significant variation.

## 3. Experimental Details

We prepared the (Lu_1/2_Nb_1/2_)*_x_*Ti_1−*x*_O_2_ ceramics with *x* values of 0.005 (LuNTO-1), 0.010 (LuNTO-2), and 0.025 (LuNTO-3) via an SSR process. The raw materials consisted of Lu_2_O_3_ (99.9% purity, St Louis, MO, USA), TiO_2_ (99.9% purity, St Louis, MO, USA), and Nb_2_O_5_ (99.99% purity, St Louis, MO, USA). The oxides were mixed via a wet ball-milling process, using ethanol as the mixing media. Details of this preparation process have previously been reported [11,15,33]. The obtained mixed powders were pressed into pellets without calcination. Finally, the samples were heated up from 30 °C at the rate of 5 °C/min, then sintered in air at 1450 °C for 6 h, and then cooled to 30 °C at the rate of 5 °C/min.

X-ray diffractometry (XRD, PANalytical, EMPYREAN) (Shanghai, China), scanning electron microscopy (SEM, FEI, QUANTA 450, Hillsboro, OR, USA), and energy-dispersive X-ray spectroscopy (EDS) were used to characterize the phase structure and surface morphologies of the sintered ceramics. The chemical states adopted by each sample were evaluated using X-ray photoelectron spectroscopy (XPS, PHI5000 VersaProbe II, ULVAC-PHI, Chigasaki, Japan) at the SUT-NANOTEC-SLRI Joint Research Facility, Synchrotron Light Research Institute (SLRI), Thailand. The XPS spectra were fitted using PHI MultiPak XPS software using a combination of Gaussian and Lorentzian equations. The sintered ceramics were further characterized using Raman spectroscopy (Bruker, Senterra II, Ettlingen, Germany). To perform the dielectric measurements, the sintered ceramics were polished to remove the surface layer before being used to form two parallel electrodes. A conductive silver paint was added to the polished ceramics to form electrodes before being heated in air at 600 °C for 0.5 h. The dielectric properties were evaluated using an impedance analyzer (KEYSIGHT E4990A, Santa Rosa, CA, USA) at a V_rms_ of 500 mV. The dielectric properties were obtained at temperatures between −60 °C and 210 °C and frequencies ranging between 40–10^7^ Hz.

## 4. Conclusions

Highly dense LuNTO ceramic microstructures were successfully prepared via an SSR method. This novel variety of GD oxide LuNTO ceramics exhibited extremely low tanδ values of approximately 0.007 and extremely high ε’ values of approximately 7.55 × 10^4^ at 10^3^ Hz. A slight reduction in the Δε’ to < ±15% was observed in the temperatures between −60 °C and 210 °C. The electrical responses exhibited by the grain, GB, and second phase particles within the microwave-dielectric LuNbTiO_6_ phase exerted a remarkable influence on the dielectric properties. The microstructure exhibited by the LuNTO ceramics consisted of semiconducting grains alongside insulating GBs and LuNbTiO_6_ phase particles. The significant increase in the ε’ exhibited by the LuNTO ceramics was attributed to the introduction of semiconducting grains as a result of the presence of the Ti^3+^ ions induced through Nb^5+^ doping, while the low tanδ value was primarily attributed to the high resistance imparted by the insulating GBs. Despite this, we conclude that the insulating LuNbTiO_6_ phase particles were not the primary cause of the reduction in the tanδ values at temperatures around 25 °C, while the temperature stability of the ε’ parameter was attributed to the introduction of additional dielectric relaxation. Because of their excellent dielectric properties, the LuNTO ceramics co-doped with a small amount of Lu^3+^/Nb^5+^ ions can be utilized toward the development of materials for applicable in ceramic capacitors.

## Figures and Tables

**Table 1:** Dielectric properties at 1 kHz and 25 °C, lattice parameters, and mean grain sizes.

Sample	Dielectric Properties			Lattice Constant (Å)		Mean Grain Size (μm)
	ε’ (25 °C)	tanδ (25 °C)	tanδ (200 °C)	*a*	*c*	
LuNTO-1	75,524	0.007	0.050	4.595(8)	2.959(5)	18.2 ± 6.9
LuNTO-2	57,137	0.028	0.057	4.599(1)	2.962(9)	15.6 ± 6.3
LuNTO-3	60,134	0.048	0.062	4.607(0)	2.974(2)	8.6 ± 2.9

## Data Availability

The data presented in this study are available in article.

## Sample Availability

Samples of the compounds are not available from the authors.
